# An Outrage on Texas!

**Published:** 1897-09

**Authors:** 


### EDITORIAL DEPARTMENT.

F. E. DANIEL, M. D., Editor.
S. E. HUDSON, M. D., Managing Editor.
A. J. SMITH. M. D., Galveston, Associate Editor.

Published Monthly at Austin, Texas, by Drs. Daniel and Hudson. Subscription price $2.00 a year in advance.

Eastern Agents: The Monihan-Fairchild Special Agency, St. Paul Building, New York City.

Official organ of the West Texas Medical Association, the Houston District Medical Association, the Austin District Medical Society, Brazos Valley Medical Association, the Galveston County Medical Society, and several others.

### AN OUTRAGE ON TEXAS!

**The Marine Hospital Service of the U. S. Seizes and Holds a State Quarantine Station in Open Violation of Law and in Face of Protests from the Governor of Texas, from the State Health Officer and from the People.—A Marine Hospital Surgeon Supercedes the State Quarantine Officer and is Performing the Functions of Quarantine at Sabine Pass.**

It will be remembered that the last Legislature was memorialized, in the name of the Texas State Medical Association, to "transfer the expense of quarantining the State to the general government," and that the question was disposed of in the Senate in these words: "The quarantine laws of Texas need no change or amendment." The same, coming up in the House, later, in connection with the appropriation for quarantine, was voted down by a vote of 85 to 11.

Notwithstanding this emphatic negative given the proposition, the supervising Surgeon General of the Marine Hospital Service is determined, it seems, to administer quarantine in Texas *nolens volens.* He has seized upon the merest pretext for interference, and has, without a shadow of authority, taken possession of the State quarantine station at Sabine Pass, superceding the veteran State quarantine officer, Dr. Perkins, who has served the State in that capacity at that point for fifteen years; and this, too, despite the energetic and emphatic protest of our Governor, and of the State Health Officer, and even of the people of Sabine Pass. If this is not a high-handed outrage, I do not know what it lacks to make it so.

The paper "On the Relation of Federal to State Quarantine," published herewith, read by State Health Officer Swearingen at the conference of State Boards of Health held at Nashville on the 18th ult., gives a clear statement of facts which led to the interference on the part of the M. H. S., as well as a little "unwritten history" which may throw additional light on the subject. The town builders at Sabine Pass invoked the aid of the M. H. S., under the pretext of "danger," and an inspector having been sent to the Pass, reported the finding of a veritable mare's nest. Whereupon the Supervising Surgeon General sent the alarming(?) telegram to the State Health Officer at Austin which is reproduced in the paper referred to, published elsewhere in this issue.

To this telegram Dr. Swearingen replied as follows:

"AUSTIN, TEXAS, July 19, 1897.

"*Surgeon Gen'l Walter Wyman, M. H. S., Washington, D. C.:*

"I intend to move Sabine pass station two miles south of present site as soon as permission is given by the War Department. The danger has been exaggerated. When infected vessels arrive at a port complete isolation is enforced.

"R. M. SWEARINGEN, S. H. O."

NOTWITHSTANDING this assurance Dr. Wyman assigned Dr. Magruder to dnty at Sabine Pass, with orders from the Secretary of the Treasury that *pratique* should be granted to no vessel without his (Magruder's) order, and that he (Magruder) should disinfect vessels, etc., or send them to Ship Island for treatment. The State Health Officer of Texas was notified of this action. It seems that the pretext for stationing Magruder there was that Dr. Perkins refused to obey verbal orders of Dr. Magruder, and to disinfect vessels under his supervision. He told Dr. M. that he could "send all vessels to Ship Island or to ——" elsewhere, but that he could neither treat them at Sabine Pass nor oversee him while he did so.

It seems there was in port at the time a schooner (the Alice) from Vera Cruz. She had discharged her ballast, and was in quarantine. Having discharged her ballast, it was inexpedient to send her to Ship Island. Hence the lock occurred. Perkins would not disinfect while Magruder was in charge of the station and overseeing him by orders from Wyman, nor would he allow

Magruder to do so at that point. This was the pretext for superceding the State officer.

In reply to the notification of Magruder's assignment, State Health Officer Swearingen wrote Dr. Wyman as follows:

AUSTIN, TEXAS, August 12th, 1897.

*Dr. Walter Wyman, Supervising Surgeon-General, Marine Hospital Service, Washington, D. C.*

DEAR SIR:—I have the honor to acknowledge the receipt of your communication of 9th inst., relating to the assumption of control by the Marine Hospital Service, of the function of quarantine at Sabine Pass. I would respectfully submit that the quarantine act of 1893 authorizes such assumption *only in case of failure or refusal of the State authorities to enforce the rules and regulations promulgated by the Treasury Department*, and I respectfully beg to assure you that at no time have I, or any of the officers of this department, ever failed, refused or neglected to enforce such rules where it has been necessary or required of us to do so. Dr. Perkins' declining to permit Dr. Magruder to exercise a supervision over him, and refusing to obey his verbal orders can not, by any construction, be said to be a breach of this act. I therefore earnestly request that you reconsider your action. Granting that you, acting upon Dr. Magruder's report of the circumstances understood it to mean such refusal, it would have been more consistent with the courtesy which has heretofore characterized the official relations of the M. H. S. and this department, if you had so written me, and given me the opportunity to correct the misapprehension. As chief health of Texas, I am responsible to the people of the State for the safe guarding of the public health, and it is not only my duty, but it has always been my pleasure, to avail myself of everything that may contribute to that end. I have therefore been careful to see that no rule or regulation promulgated by the Secretary of the Treasury has been neglected. I trust, for the sake of harmony, as well as for the best interests of the service that you may see this matter in its true light, and hasten to recall Dr. Magruder. His presence there under the circumstances, cannot but prove a source of friction, being, as I have pointed out, unauthorized by any reasonable interpretation of the act referred to, and altogethər unnecessary.

I have just returned from Sabine Pass where I spent twenty-four hours. The initiative has been taken for the speedy construction of a new station about two miles from the present site, and the work will be pushed to a speedy completion.

I expect to attend the conference of State Boards of Health, at Nashville on 18th inst., and to read a paper which I have prepared. It is on "The Relation of Federal to State Quarantine," and I would be much gratified if you can make it convenient to be present, Very truly yours,

R. M. SWEARINGEN, M. D.
State Health Officer.

Governor Culberson also wrote to Mr. Gage, Secretary of the Treasury, protesting most emphatically against the unwarrantable assumption of control of a State quarantine, and asked that the wrong be speedily righted.

To the date of this writing—August 31, no reply to either of the above protests has beeen received! Insult added to injury.

Meantime the interests of those who invoked Federal interference did not tally with those of lumber shippers and others who were suffering during the tie-up of the Alice, and representatives of the latter appealed to the Governor to "*make* Perkins allow Magruder to run the station." Letters and telegrams from the owners of the Alice, addressed to the Governor and to the State Health Officer received the following answer.

The Governor wrote as follows:

AUSTIN, TEXAS, August 13, 1897.

*Mr. J. F. Keith, Beaumont, Texas.*

"DEAR SIR:—The collector of the port at Sabine Pass has instructions from the United States government to not recognize *pratique* granted vessels by State Quarantine officers. I have no authority over the Marine Hospital Surgeon who has taken possession of Sabine Pass. Dr. Perkins would conform to the rules and requirements of the State service if not interfered with.

[Signed] C. A. CULBERSON."

Dr. Swearingen replied to Mr. Keith as follows:

QUARANTINE DEPARTMENT,
AUSTIN, TEXAS, August 14, 1897.

*Mr. J. F. Keith, Beaumont, Texas.*

"DEAR SIR:—In answer to your telegram of to-day to the Governor asking that the Marine Hospital Surgeon be permitted to stay at the station to 'see that the rules are obeyed,' I beg to say: We have pledged the government to carry out the instructions promulgated by the Secretary of the Treasury—[as required by Sec. 3, of the Federal Quarantine law of 1893.] To allow an inspector to stay there as a guard to see that we are honest would be an insult to any officer. I certainly will not require Dr. Perkins to submit to such espoignage. I regret the state of affairs at Sabine Pass but am in no way responsible for it.

[Signed] R. M. SWEARINGEN."

Finding,—as is usually the case,—that innocent parties were suffering in consequence of this high-handed meddling with the State affairs, Dr. Perkins finally agreed to step aside and permit Dr. Magruder to perform the functions of his office, and so notified him on the 20th of August. Notwithstanding which, Dr. Magruder *declined to officiate* until he had received explicit and additional orders from Wyman to go ahead and disinfect the schooner Alice. Had he promptly done so on receiving Dr. Perkins' permission, the vessel, which was under contract to deliver ties in Mexico in August, would have been enabled to make good her contract; as it is, she forfeits it, and her owners lose several hundred dollars.

In addition to the protest of the Governor and of the State Health Officer, a large number of the citizens of Sabine Pass and Beaumont met and drew up the following remonstrance and protest against the action of the Marine-Hospital Service,—representing that the presence, at Sabine Pass, of an inspector of the Marine-Hospital Service, under the circumstances, was embarrassing and ruinous to the business interests of the place, and respectfully petitioning the Chief for his speedy removal, and a restoration of the *status quo.*

PROTEST OF BUSINESS MEN.

SABINE PASS, TEXAS, August, 1897.

*To the Surgeon-General, M. H. S., Washington, D. C.:*

We, the undersigned business men of Sabine Pass, do hereby protest against the actions of your subordinate, Dr. Magruder.

Dr. Perkins, our State health officer, has agreed and conceded to Dr. Magruder everything in reason, but of no avail. Dr. Magruder will do nothing himself, nor will he let Dr. Perkins do anything, refusing to let vessels enter the custom house; and by his arbitrary actions has worked a great hardship and damage to our shipping interest.

We therefore protest against such actions as ruining our port, and pray you to give immediate relief by recalling Magruder.

(Signed by the business men of the port.)

And all this tempest in a teapot for the sole purpose of gratifying Dr. Wyman's ambition to run the whole quarantine. There has not been a shadow of danger at Sabine Pass; so we are assured by the State Health Officer. Dr. Wyman, in taking this step, has clearly transcended his authority,—as pointed out in Dr. Swearingen's paper. In short, it is a case of might vs. right, and is in keeping with the general tendency to centralization, which has cropped out occasionally, lately; notably in Illinois, during the strike, when Cleveland sent U. S. troops to that State over the protest of the governor; and, more recently, in Kansas, in the matter of the Federal Court suspending a State law regulating insurance.

If Dr. Wyman, in the face of the above many assurances that he is intruding where he is not wanted nor welcome,—in face of protest from the Governor, from the State Health Officer, and, finally, from the people,—persists in his determination, he is certainly very thick-skinned. If he is upheld in this course by the courts,—for it will finally come to a question of the constitutionality of the act of February, 1893 (see Dr. Swearingen's paper), it will be the death blow to the question of the rights of a State to administer its own police,—a question once very dear to every Southern man.

In this connection the JOURNAL has to record the fact that Charleston, the capital of old South Carolina, the cradle of State's Rights, the Storm Center of Secession, is the *first* city to surrender the principle. Her municipal government has asked the U. S. government to take her quarantine off her hands. Poor old Charleston! How are the mighty fallen! The present generation of her people are surely not the legitimate descendants of those who fired on Sumpter in defense of that same principle! We hope she may not suffer the fate of Brunswick, Georgia.

What the final result will be in reference to the Texas quarantine remains to be seen. Meantime Dr. Perkins is on duty, with his work taken off his hands. We will see. That the State is right, no fair-minded person acquainted with the facts will deny. That Wyman has charge of one of her quarantine stations without the consent of the State and without warrant of law is equally patent. He has seized it upon a mere pretext.

The JOURNAL predicts that he will make haste to reconsider his action, and will relieve the State of the presence of Dr. Magruder, which is as embarrassing as it is unnecessary.

